# Prevalence of Branch Retinal Vein Occlusion in the Latin Population: Insights Into Clinical Epidemiology

**DOI:** 10.7759/cureus.109215

**Published:** 2026-05-19

**Authors:** Mario Leon Meza, Braulio Hernán Velasco-Sepúlveda, Jorge Gerardo Morales Navarro, Miguel Angel Hernandez-Delgado, Federico Graue Wiechers, Daniel Cortes Muñoz

**Affiliations:** 1 Retina, Instituto de Oftalmología Fundación Conde de Valenciana, Mexico City, MEX; 2 Ophthalmology, Instituto de Oftalmología Fundación Conde de Valenciana, Mexico City, MEX

**Keywords:** branch retinal vein occlusion, macular edema, neovascular glaucoma, retinal injury, retinal occlusions

## Abstract

Introduction

Branch retinal vein occlusion (BRVO) is the most common form of retinal vein occlusion and a major cause of vision loss. Although its prevalence and clinical features have been well documented globally, epidemiological data from the Mexican population remain limited.

Purpose

This study aimed to characterize the prevalence, clinical features, risk factors, and complications of BRVO in a Mexican population treated at a tertiary ophthalmology center.

Methodology

A retrospective, descriptive, observational study was conducted at the Instituto de Oftalmología* *Fundación Conde de Valenciana in Mexico City, Mexico. Medical records of patients diagnosed with BRVO between January 2012 and January 2022 were reviewed. Inclusion criteria required a minimum follow-up of six months. Patients with incomplete records or ocular comorbidities other than glaucoma were excluded. Descriptive statistics and subgroup comparisons were performed using IBM SPSS Statistics for Windows, V. 29.0 (IBM Corp., Armonk, NY, USA), with statistical significance set at p < 0.05.

Results

Out of 1,639 reviewed clinical records, 211 patients met the inclusion criteria, yielding a BRVO prevalence of 2.94 cases per 1,000 patients (0.294%). The mean age was 63 years, and 111 (52.61%) patients were female. Hypertension was present in 96 (45.50%) cases, diabetes was present in 19 (9.00%), and both conditions were present in 33 (15.64%). Glaucoma was found in 35 (16.59%) patients. The most common site of occlusion was the superotemporal quadrant (100 (47.39%)). Complications developed in 163 (77.25%) cases within the first three months, with macular edema (151 (71.56%)) being the most frequent, followed by retinal neovascularization (28 (13.27%)) and neovascular glaucoma (5 (2.37%)). Macular edema was predominantly associated with superotemporal BRVO and often presented within the first month. Macular ischemia was rare (1 (0.47%)) but was associated with poor visual outcomes.

Conclusion

This study provides the first detailed epidemiological insight into BRVO in a Mexican population, with findings broadly consistent with the international literature. Hypertension and glaucoma were the most common systemic and ocular risk factors, respectively. Macular edema was the leading cause of visual morbidity and developed early in the disease course. These findings underscore the importance of early diagnosis and intervention, particularly in patients with high-risk profiles. Comprehensive systemic evaluation and prompt ocular treatment, especially for macular edema and neovascularization, are essential to optimize visual outcomes and reduce long-term complications.

## Introduction

Retinal vein occlusion (RVO) is the second most common retinal vascular disorder after diabetic retinopathy and represents a major cause of visual impairment worldwide. First described by Leber, RVO is traditionally classified into two main types: central retinal vein occlusion (CRVO), which involves the obstruction of the central retinal vein, and branch retinal vein occlusion (BRVO), which affects one of its peripheral branches. Among these, BRVO is more prevalent, occurring approximately three times more frequently than CRVO [1].

BRVO most commonly affects the superotemporal quadrant of the retina. This predilection may be related to the higher number of arteriovenous crossings in that region or to the fact that extramacular occlusions, particularly those occurring nasally, often remain asymptomatic and therefore go undiagnosed [2].

Several systemic conditions are strongly associated with RVO, particularly in individuals older than 60 years, regardless of sex. The most important risk factors include hypertension, diabetes mellitus, and atherosclerosis. Glaucoma is another important contributor, increasing the risk of RVO by approximately 3.3-fold. In patients younger than 50 years, less common etiologies such as thrombophilia, inflammatory disorders, viral infections, or substance abuse should be considered during the diagnostic evaluation [3].

The pathogenesis of BRVO involves all three components of Virchow's triad: venous stasis, endothelial injury, and hypercoagulability. These factors often converge at arteriovenous crossings, where a thickened arterial wall may compress the adjacent vein, leading to turbulent flow, endothelial damage, and subsequent thrombus formation. The resulting vascular obstruction can lead to complications including macular edema, macular ischemia, subretinal fibrosis, epiretinal membrane formation, retinal atrophy, and irreversible visual loss. Before the introduction of anti-vascular endothelial growth factor (anti-VEGF) therapies, approximately 36% of eyes with extensive retinal ischemia, defined as at least five disc areas of nonperfusion on fluorescein angiography, developed neovascularization involving the retina or optic disc [4].

The aim of this study was to describe the prevalence and clinical characteristics of BRVO in patients treated at the Instituto de Oftalmología Fundación Conde de Valenciana, a tertiary care ophthalmology center in Mexico. 

## Materials and methods

This retrospective, descriptive, observational study was conducted at the Instituto de Oftalmología Fundación Conde de Valenciana in Mexico City, Mexico, and was based on a review of the clinical records of patients diagnosed with BRVO between January 2012 and January 2022.

Inclusion criteria

Eligible patients were those diagnosed with BRVO and treated at the aforementioned institute between January 2012 and January 2022, with a minimum follow-up period of six months.

Exclusion criteria

Patients were excluded in cases of incomplete medical records, follow-up of less than six months, or the presence of ocular comorbidities other than glaucoma that could affect the interpretation of clinical findings and outcomes.

Descriptive statistics were used to summarize qualitative variables as frequencies and percentages and quantitative variables as measures of central tendency and dispersion. Normality was assessed using the Kolmogorov-Smirnov test. Quantitative variables were expressed as means. Subgroup comparisons based on categorical variables were performed using Pearson's chi-squared test and independent t-tests. Statistical significance was set at p < 0.05. All analyses were performed using IBM SPSS Statistics for Windows, V. 29.0 (IBM Corp., Armonk, NY, USA). 

All study procedures adhered to the tenets of the Declaration of Helsinki and were approved by the institutional ethics committee, which waived the requirement for informed consent because of the retrospective nature of the study. 

## Results

A total of 1,639 clinical records were identified during the 10-year study period, and 211 patients met the inclusion criteria. The prevalence of BRVO was 2.94 cases per 1,000 patients (0.294%). Among the included cases, 111 (52.61%) were women. The mean age was 63 years, and the mean best-corrected visual acuity at diagnosis was 1.05 logarithm of the minimum angle of resolution (logMAR) (Table 1). 

**Table 1:** Baseline demographic and clinical characteristics BRVO: branch retinal vein occlusion; logMAR: logarithm of the minimum angle of resolution

Variable	Value
Total clinical records reviewed	1,639
Included patients with BRVO	211
BRVO prevalence	2.94 per 1,000 patients (0.294%)
Female sex	111 (52.61%)
Male sex	100 (47.39%)
Mean age at diagnosis	63 years
Mean best-corrected visual acuity at diagnosis	1.05 logMAR

The most common systemic comorbidities were hypertension in 96 (45.50%) patients and diabetes mellitus in 19 (9.00%) patients. Both hypertension and diabetes mellitus were present in 33 (15.64%) patients, and dyslipidemia was identified in 11 (5.21%) patients. Twenty-one (9.95%) patients had a history of smoking. Among ocular comorbidities, glaucoma was present in 35 (16.59%) patients, previous contralateral RVO in 12 (5.69%), nonproliferative diabetic retinopathy in 11 (5.21%), and pars planitis in one (0.47%) patient (Table 2). 

**Table 2:** Systemic and ocular comorbidities

Variable	N (%)
Hypertension	96 (45.50%)
Diabetes mellitus	19 (9.00%)
Hypertension and diabetes mellitus	33 (15.64%)
Dyslipidemia	11 (5.21%)
Smoking history	21 (9.95%)
Glaucoma	35 (16.59%)
Previous contralateral retinal vein occlusion	12 (5.69%)
Nonproliferative diabetic retinopathy	11 (5.21%)
Pars planitis	1 (0.47%)

In terms of location, the most common site was the superotemporal quadrant, observed in 100 (47.39%) cases, followed by the inferotemporal quadrant in 75 (35.55%) cases. Nasal involvement, both superonasal and inferonasal, was observed in four (1.90%) cases each. Inferior hemiretinal occlusion was present in 17 (8.06%) cases and superior hemiretinal occlusion in 11 (5.21%) cases. Considering all complications, 163 (77.25%) occurred within the first three months (Table 3). 

**Table 3:** Distribution of BRVO by occlusion site BRVO: branch retinal vein occlusion

Occlusion site	N (%)
Superotemporal quadrant	100 (47.39%)
Inferotemporal quadrant	75 (35.55%)
Superonasal quadrant	4 (1.90%)
Inferonasal quadrant	4 (1.90%)
Inferior hemiretinal occlusion	17 (8.06%)
Superior hemiretinal occlusion	11 (5.21%)

The main complications observed were macular edema in 151 (71.56%) patients and retinal neovascularization in 28 (13.27%) patients. Among patients who developed macular edema, 127 (84.11%) presented within the first month. Of the five (2.37%) patients who developed neovascular glaucoma, three (60.00%) presented within the first three months. As the most common complication, macular edema was most frequently associated with superotemporal BRVO as seen in 68 (32.22%) cases, followed by inferotemporal BRVO in 56 (26.54%) cases. Retinal neovascularization was also most commonly associated with superotemporal BRVO, observed in 12 (5.68) cases, followed by inferotemporal BRVO in seven (3.31%) cases (Table 4).

**Table 4:** Main complications and timing of presentation *Percent calculated using 151 patients with macular edema. **Percent calculated using five patients with neovascular glaucoma.

Variable	N (%)
Any complication within the first 3 months	163 (77.25%)
Macular edema	151 (71.56%)
Retinal neovascularization	28 (13.27%)
Neovascular glaucoma	5 (2.37%)
Macular ischemia	1 (0.47%)
Tractional retinal detachment	2 (0.95%)
Macular edema presenting within the first month*	127 (84.11%)
Neovascular glaucoma presenting within the first 3 months**	3 (60.00%)

Macular ischemia was identified in only one (0.47%) patient and was associated with a significant negative impact on final visual acuity. Neovascular glaucoma was present in five (2.37%) patients, and most cases developed within the first three months (3 (60.00%)). Tractional retinal detachment occurred in two (0.95%) patients (Tables 4-5).

**Table 5:** Distribution of major complications according to BRVO location* *Percentages do not sum to 100% because additional cases were distributed across other occlusion sites. BRVO: branch retinal vein occlusion

Complication	Superotemporal BRVO, N (%)	Inferotemporal BRVO, N (%)
Macular edema (n = 151)	68 (45.03%)	56 (37.09%)
Retinal neovascularization (n = 28)	12 (42.86%)	7 (25.00%)

## Discussion

This study provides relevant data on the epidemiology, clinical characteristics, risk factors, and complications of BRVO in a population treated at a tertiary ophthalmic referral center in Mexico. In contrast to the prevalence reported in international studies such as the Beaver Dam Eye Study and the Blue Mountains Eye Study, the prevalence of BRVO in our population was lower [5,6]. This difference may be explained by variations in comorbidity management, access to health care, or demographic characteristics within the study population. 

The mean age at diagnosis was 63 years, which is consistent with previous reports showing a higher incidence in older adults. In terms of sex distribution, BRVO was slightly more common in women than in men. 

The three most common systemic risk factors were systemic arterial hypertension (96 (45.50%)), diabetes mellitus (19 (9.00%)), and dyslipidemia (11 (5.21%)). These findings are generally consistent with the literature. In a systematic review, O'Mahoney et al. reported a pooled prevalence of 67.7% for systemic hypertension, 15.7% for diabetes mellitus, and 40.4% for hyperlipidemia [7]. In our study, diabetes mellitus was more prevalent than dyslipidemia, which may be explained by the underestimation or underdiagnosis of dyslipidemia in this population. Furthermore, the low prevalence of thrombophilia suggests that these disorders should be specifically investigated in younger patients, such as those in our study whose ages were well below the average age at RVO diagnosis.

With a prevalence of 35 (16.59%) patients, glaucoma was the most common ocular comorbidity, reinforcing its role as a recognized risk factor for BRVO. The rare case of a young patient with intermediate uveitis highlights the need to investigate less common etiologies in atypical patients. More severe branch occlusions involving the inferotemporal arcade tended to be associated with worse visual acuity at presentation. Overall, the superotemporal arcade was the most commonly affected site in BRVO, followed by the inferotemporal arcade.

In the present study, the prevalence of BRVO was 2.94 cases per 1,000 patients. This was higher than that reported in some similar studies. The Beaver Dam Eye Study reported a prevalence of 0.4-0.9 per 100 patients, with an overall prevalence of 0.6% [6]. The Blue Mountains Eye Study reported a prevalence of 1.3-1.9 per 1,000 patients, with an overall prevalence of 1.6%. Rogers et al. reported a prevalence of 5.7-8.3 per 1,000 in the Hispanic population in 2013 [8].

Regarding the distribution of occlusion sites, Thapa et al. reported that 66% of cases involved the superotemporal arcade [9]. Similarly, the Blue Mountains Eye Study found a comparable prevalence of 64% in the same vascular region [6]. In our study, the superotemporal arcade was involved in 100 (47.39%) cases. With respect to inferotemporal branch occlusions, the Blue Mountains Eye Study reported a prevalence of 20%. In contrast, hemiretinal vein occlusions appear to be less common. A study conducted in India by Kadam et al. reported a prevalence ranging from 0.5% to 2% [10]. In our findings, the prevalence was 11 (5.21%) for superior hemiretinal vein occlusion and 17 (8.06%) for inferior hemiretinal vein occlusion.

With macular edema present in 151 (71.56%) cases at the time of diagnosis, it was the most frequent complication, highlighting its significance as the leading cause of vision loss in BRVO. Macular ischemia and neovascularization were less common complications; however, the latter may have been underdiagnosed because some general practitioners may have been unfamiliar with its evaluation. Given that these complications are associated with increased VEGF levels, prompt administration of intravitreal anti-VEGF therapy is crucial.

Macular edema was primarily treated with intravitreal anti-VEGF therapy, with favorable outcomes observed with agents such as ranibizumab and aflibercept. Panretinal photocoagulation was performed in all cases with neovascularization, whereas neovascular glaucoma and tractional retinal detachment were managed surgically. 

Given that poorer visual acuity at presentation was associated with worse final visual outcomes, early evaluation and treatment are essential. Hemiretinal occlusions, which are associated with greater ischemia and macular edema, likewise showed worse visual outcomes both at presentation and at the end of follow-up. 

This study has several limitations. First, its retrospective design may have introduced selection and information bias, as the analysis depended on the accuracy and completeness of medical records. Second, because the study was conducted at a single tertiary referral center, the findings may not be fully generalizable to the broader Mexican population or to primary and secondary care settings. Third, some systemic comorbidities and ocular findings may have been underdiagnosed or underreported, particularly when documentation was incomplete or evaluation practices varied among clinicians. In addition, the requirement for a minimum follow-up of six months may have excluded patients with shorter follow-up and introduced selection bias. Finally, treatment approaches and diagnostic methods may have changed over the 10-year study period, which could have influenced the frequency of detected complications and clinical outcomes.

## Conclusions

BRVO represents a significant condition in the studied population, exhibiting clinical and epidemiological patterns consistent with international findings. Timely recognition and management of its complications, particularly macular edema and neovascularization, are crucial for preserving visual function. These results highlight the need for close monitoring during the early stages after diagnosis to detect sight-threatening complications such as posterior segment neovascularization and neovascular glaucoma. Chronic or persistent macular edema should prompt evaluation for underlying macular ischemia. These findings underscore the importance of a multidisciplinary approach to improve diagnostic accuracy and optimize treatment outcomes, particularly in younger patients or those with atypical risk factors. 
